# Effect of relative masker levels on speech recognition in two-talker maskers with varying perceptual similarity to the target speech

**DOI:** 10.1121/10.0019955

**Published:** 2023-07-05

**Authors:** Mathew Thomas, John J. Galvin, Qian-Jie Fu

**Affiliations:** 1 Department of Head and Neck Surgery, David Geffen School of Medicine, University of California, Los Angeles, Los Angeles, California, 90095, USA; 2 House Institute Foundation, Los Angeles, California 90057, USA mathewthomas@g.ucla.edu, jgalvin@hifla.org, qfu@mednet.ucla.edu

## Abstract

Speech recognition thresholds were measured as a function of the relative level between two speech maskers that differed in perceptual similarity from the target. Results showed that recognition thresholds were driven by the relative level between the target and perceptually similar masker when the perceptually similar masker was softer, and by the relative level between the target and both maskers when the perceptually similar masker was louder. This suggests that effectiveness of a two-talker masker is primarily determined by the masker stream that is most perceptually similar to the target, but also by the relative levels between the two maskers.

## 1. Introduction

Masked-sentence recognition is particularly poor when the masker is composed of two competing talkers, a finding that is attributed to significant amounts of informational masking introduced (e.g., Freyman *et al.*, 2004; Rosen *et al.*, 2013). Informational masking tends to be largest when the target and masker speakers are perceptually similar (e.g., Kidd *et al.*, 2016; Calandruccio *et al.*, 2017; Thomas *et al.*, 2021). For two-talker maskers, the component masker sentences may vary with respect to perceptual similarity relative to the target. An intelligible competing talker having the same sex as the target talker is likely to be perceptually similar to the target, while an unintelligible competing talker (e.g., non-native or time-reversed) or a competing talker with a different sex from the target is likely to be perceptually dissimilar to the target. Calandruccio *et al.* (2017) evaluated sentence recognition in normal-hearing (NH) adults in the presence of two-talker maskers. The perceptual similarity between the target and each of the two masker streams was varied to include combinations of intelligible (native language) or unintelligible (non-native or time-reversed) speech. Target sentence recognition with one intelligible and one unintelligible masker was only marginally better than with two intelligible maskers. The authors suggested that the effectiveness of a two-talker masker is dominated by the masker stream that is most perceptually similar to the target, with little contribution from the less similar masker. Similar findings were also reported when the masker sex was the same or different from the target (e.g., Chen *et al.*, 2020; Zhang *et al.*, 2020; Thomas *et al.*, 2021). For example, Thomas *et al.* (2021) found little difference in speech recognition thresholds (SRTs) with two-talker maskers when the masker sex for both maskers or for one of the two maskers was the same as the target.

A perceptually similar masker stream likely produces more informational masking than a perceptually dissimilar masker stream (e.g., Kidd *et al.*, 2016). The perceptual similarity between the target and the same-sex masker stream can be due in part to similar talker F0s. Similar talker F0s (e.g., same-sex maskers) would also be expected to produce more energetic masking than would dissimilar F0s (different-sex maskers). However, the difference in energetic masking between same- and different-sex masker streams is likely minimal. Thomas *et al.* (2021) found no significant difference in SRTs between reversed same-sex and reversed different-sex masker streams (i.e., less informational masking due to the lack of lexical cues) for one-talker and two-talker maskers. This suggests that differences in masker talker F0s may not produce any meaningful difference in energetic masking between same- and different-sex masker streams. For two-talker maskers, replacing a perceptually similar masker stream with a dissimilar stream appears to have little effect on SRTs (Calandruccio *et al.*, 2017; Thomas *et al.*, 2021), suggesting no significant reduction in the degree of informational masking.

In most previous studies with two-talker maskers, the root mean square (RMS) amplitude is typically the same for each component masker. However, the relative levels across multiple background talkers are rarely the same and vary over time during daily conversation. To our knowledge, few studies have investigated the effects of relative level differences between two masker streams on speech recognition. Varying the relative levels of two maskers may affect the degree of informational masking, depending on the perceptual similarity between the two masker streams. When two maskers were both perceptually similar to the target (i.e., same-sex), Brungart *et al.* (2001) found that speech performance depended almost exclusively on the relative level between the target and the combined maskers, at least for the relative levels tested (−6 dB to 6 dB). The authors suggested that relative masker levels may not matter when two maskers are perceptually similar to the target. When only one of the two maskers is perceptually similar to the target, speech performance may be affected by the relative level difference between maskers, given the predominance of the masker stream that is most perceptually similar to the target. Brungart *et al.* (2001) found that speech performance depends on the individual levels of the component maskers and the overall SNR relative to the combined maskers. However, it is unclear how much the less dominant masker stream may contribute, especially when the level of the more similar stream is lower than that of the dissimilar stream.

The goal of the present study was to evaluate the effects of relative masker levels for two-talker maskers where one stream was perceptually similar to the target and the other stream was perceptually different from the target. SRTs were measured as a function of relative levels of the two speech maskers, ranging from −9 to +9 dB in 3 dB steps. Masked SRTs were also measured in the presence of one male talker or one female talker alone (one-talker masker) as a control condition. We expected that SRTs with the two-talker maskers will be significantly higher (poorer) than with the one-talker maskers. If varying the relative levels between the two maskers does not influence the degree of informational masking, SRTs would be expected to be determined by the relative level between the target and the combined maskers, with the dissimilar masker largely producing energetic rather than informational masking. We expected that the data would provide useful insights regarding the effect of relative level between multiple speech maskers on the degree of informational masking.

## 2. Methods

### 2.1 Participants

Twelve NH listeners (7 females, 5 males) participated in this study (mean age at testing = 30.1 yrs; range = 20–64 yrs). All participants had pure tone thresholds <25 dB HL at all audiometric frequencies between 250 and 8000 Hz. In compliance with the ethical standards for human participants, written informed consent was obtained from all participants before proceeding with any of the study procedures. This study was approved by the Institutional Review Board at the University of California, Los Angeles (UCLA).

### 2.2 Test materials

The matrix-style test materials were drawn from the Sung Speech Corpus (Crew *et al.*, 2015, 2016), and consisted of 50 words from five categories (name, verb, number, color, and object), each of which contained 10 monosyllable words. Target sentences were generated by always selecting the name “John” (the target sentence cue word), and then a random word from the 10 words in each of the remaining four categories was selected to complete the sentences. All 50 words for the target sentence were produced by a male talker; the mean fundamental frequency (F0) across all 50 words was 106 Hz. Similarly, two different masker sentences were generated by randomly selecting words from each of the categories. For each masker sentence, words were randomly selected to be different (mutually exclusive) from the target sentence as well as from the other masker sentence. Thus, during each test trial, target and masker sentences were comprised of completely different words. Masker sentences were produced by a different male talker (mean F0: 97 Hz) or by a female talker (mean F0: 157 Hz). As such, the male masker was considered to be perceptually similar to the target and the female masker was considered to be perceptually dissimilar to the target. Note that the duration of the words used to generate the target and masker sentences varied slightly across categories and talkers. As such, after generating the target and masker sentences, the masker sentence duration was normalized in real-time to have the same duration as the target without affecting pitch using SoundTouch software (https://www.surina.net/soundtouch/).

### 2.3 Test conditions

Masked SRTs, defined as the SNR between the target and both masker sentences that produces 50% correct recognition of target keywords in sentences, were adaptively measured using a procedure similar to the coordinate response measure test from Brungart *et al.* (2001). Two target keywords (randomly selected from the number and color categories) were embedded in a five-word carrier sentence uttered by the male target talker. The name “John” was used to cue listeners to the target sentence. All words in the target and masker sentences were randomly selected to be different (mutually exclusive). During testing, listeners were asked to identify the number and color keywords from a closed set of 10 responses; the responses for the name, verb, and object categories were greyed out. Recognition of the target keywords was measured in the presence of two masker sentences (two-talker masker). One masker sentence was produced by a different male talker and by a female talker. The relative levels of the male and female masker speech streams were systematically changed from −9 to +9 dB in 3 dB steps (i.e., −9, −6, −3, 0, 3, 6, and 9 dB). Masked SRTs were also measured in the presence of one male talker and one female talker alone (one-talker masker) as a control condition.

### 2.4 Testing procedures

Stimuli were generated and diotically delivered to headphones (Sennheiser HDA 200, Old Lyme, CT) via an audio interface (Edirol UA-25, Santa Clarita, CA) connected to a mixer (Mackie 402, Bothell, WA). Due to the expected wide range in SRTs, a fixed overall presentation level was used instead of a fixed target level to avoid loud presentation levels with highly negative SNRs. For the two-talker maskers, the male and female maskers were first combined at the experimental relative level, and then globally adjusted according to the designated SNR between the target sentence and the combined two-talker masker sentences. For each test trial, after the target and masker sentences were combined, the overall presentation level was adjusted to be 65 dBA.

Masked SRTs were first measured with the male and female maskers alone (i.e., one-talker masker) using an adaptive procedure (1-up/1-down). The initial SNR was 10 dB. For each test trial, participants were instructed to listen to the target sentence (produced by the male target talker and cued by the name “John”) and then click one of the 10 response choices for each of the number and color categories; no other selections could be made for the remaining categories, which were greyed out. If the participant correctly identified both keywords, the SNR was reduced by 4 dB (initial step size); if the participant did not correctly identify both keywords, the SNR was increased by 4 dB. After two reversals, the step size was reduced to 2 dB. The SRT for each test run was calculated by averaging the last six reversals in SNR. In the adaptive procedure, if there were fewer than six reversals within 20 trials, the test run was discarded and another run was executed; however, no test runs needed to be discarded in the present study. Two to three test runs were completed for each condition and the SRT was averaged across runs. The procedures were the same for the two-talker maskers. The order of the masker level conditions (−9, −6, −3, 0, 3, 6, and 9 dB) was randomized within and across participants.

## 3. Results

**Fig. 1.**
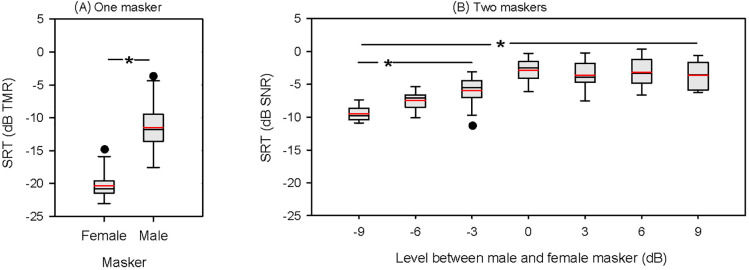
(A) Boxplots of SRTs with the female or male masker alone (one-talker maskers). (B) Boxplots of SRTs with the two-talker masker as a function of the relative levels of the male and female masker. In all panels, the black line shows the median, the red line shows the mean, the whiskers show the 10th and 90th percentiles, and the circles show outliers. The asterisks indicate Bonferroni-adjusted significant differences (p < 0.05).

Figure 1(A) shows boxplots of SRTs with the female or male masker alone. Mean SRTs were −20.3 ± 2.3 dB SNR with the female masker, and −11.5 ± 4.1 dB SNR for the male masker. A paired t-test showed that SRTs were significantly lower (better) with the female than with the male masker [t(11) = −9.615, p < 0.0001].

Figure 1(B) shows boxplots of SRTs with two-talker maskers as a function of the male/female relative level (M/F level); for negative numbers, the long-term RMS level was greater for the female than for the male masker, and for positive numbers, the long-term RMS level was greater for the male than for the female masker. Mean SRTs were −9.5 ± 1.4, −7.5 ± 1.5, −6.0 ± 2.0, −2.9 ± 1.9, −3.6 ± 2.3, −3.2 ± 2.3, and −3.6 ± 2.1 dB SNR for the −9, −6, −3, 0, 3, 6, and 9 dB M/F level conditions, respectively. A repeated measure analysis of variance (RM ANOVA) was performed on the SRT data, with M/F level (−9, −6, −3, 0, 3, 6, and 9 dB) as the factor. Results showed a significant effect for masker level [F(6, 66) = 51.6, p < 0.001]. *Post hoc* Bonferroni pairwise comparisons showed that SRTs were significantly lower (better) for negative M/F levels than for relative levels ≥ 0 dB, and significantly lower for −9 dB than for −3 or −6 dB (p < 0.05 for all comparisons); there was no significant difference between −3 and −6 dB, or among levels ≥ 0 dB.

**Fig. 2.**
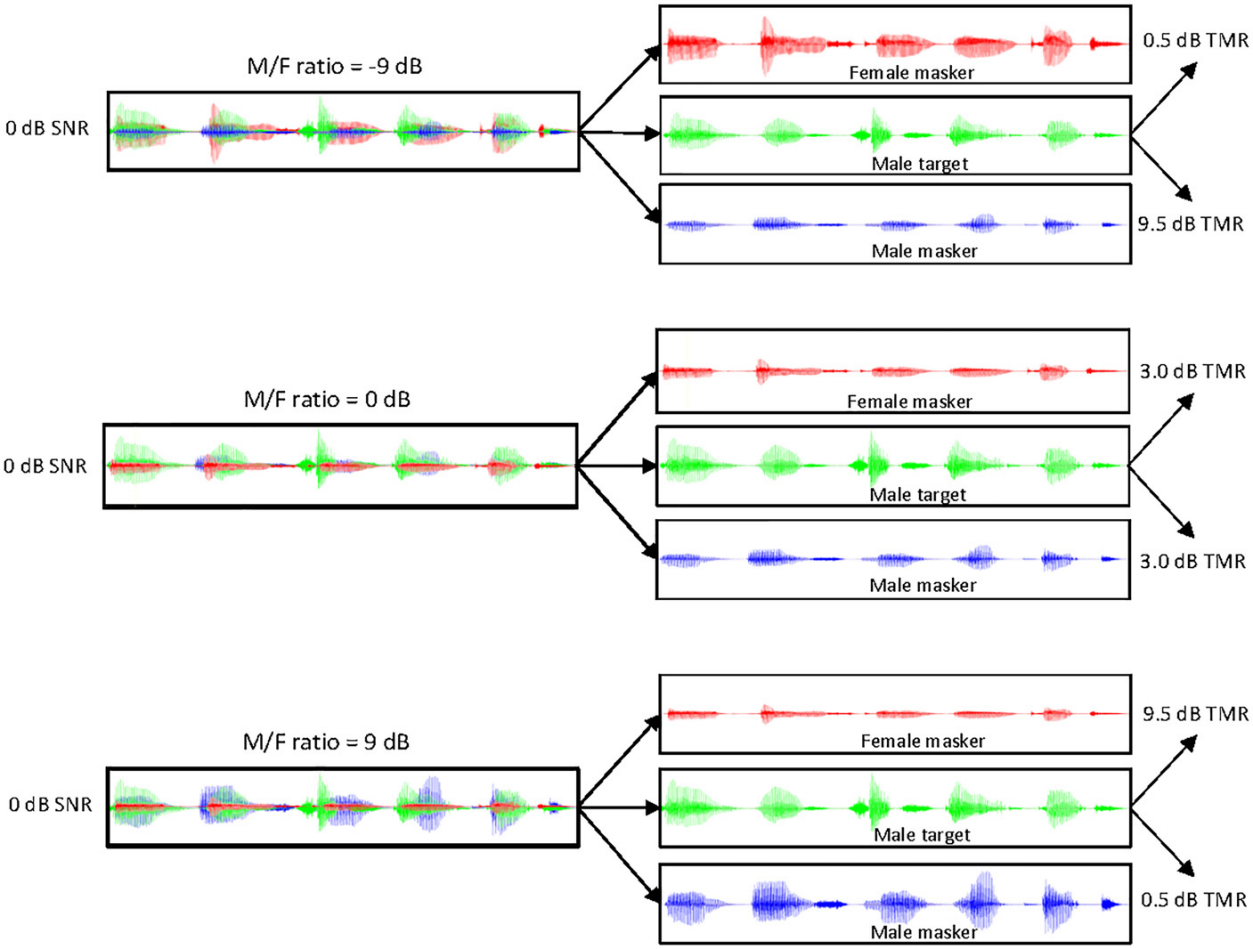
Illustration of target and masker waveforms for different M/F level conditions in terms at 0 dB SNR. The left panels show the male target (green) with the two-talker masker, composed of the female (red) and male maskers (blue). The relative levels of the male and female masker level increase from −9 to 0 to 9 dB. The right panels show the effective TMR between the target and the component female and male maskers.

Figure 2 illustrates waveforms of the target and two masker sentences at 0 dB SNR for different M/F levels (−9, 0, and 9 dB). In all panels, the same target sentence is shown in green, the female masker sentence is shown in red, and the male masker sentence is shown in blue. The overall SNR between the target and the combined two-talker masker sentences was 0 dB. When the M/F level was −9 dB, the TMR was 0.5 and 9.5 dB between the target and the female and male component masker sentence, respectively. When the M/F level was 0 dB, the target-to-masker ratio (TMR) between the target and the male component masker sentence was 3 dB between the target and the female and male component masker sentences. When the M/F level was 9 dB, the TMR was 9.5 and 0.5 dB between the target and the female and male component masker sentences, respectively.

**Fig. 3.**
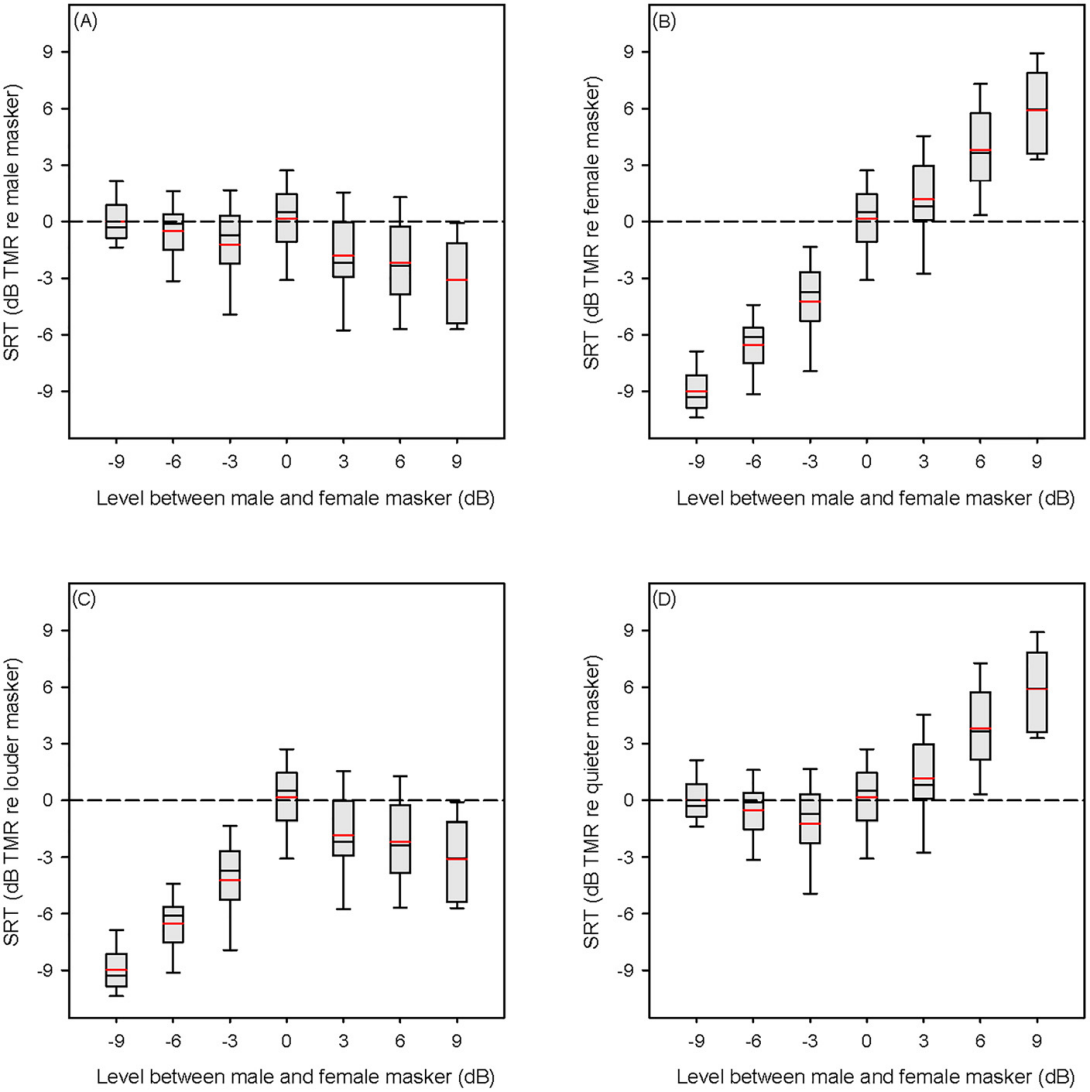
Boxplots of TMR-based SRTs as a function of the level between the male and female masker sentence, relative to the (A) male, (B) female, (C) louder, (D) quieter component masker alone. The black line shows the median, the red line shows the mean, the whiskers show the 10th and 90th percentiles.

To observe the contribution of the perceptually similar (male) component masker sentence to the two-talker SRTs [Fig. 1(B)], SRTs were recalculated in terms of the effective TMR between the target and the male component masker sentence alone at each of the M/F levels (i.e., TMR-based SRTs). Figure 3(A) shows mean TMR-based SRTs relative to the male component masker alone at different M/F levels. For comparison purposes, Figs. 3(B)–3(D) show mean TMR-based SRTs relative to the female component masker, to the louder component masker, and to the quieter component masker alone, respectively. Relative to the male component masker alone [Fig. 3(A)], mean SRTs were 0.15 dB TMR when the male and female masker sentences had the same RMS level (i.e., M/F level = 0 dB). SRTs were generally unchanged when the male masker sentence was softer than female masker sentence (i.e., M/F levels < 0 dB) but were better when the male masker sentence was louder than the female masker sentence (i.e., M/F levels > 0). An RM ANOVA was performed on the TMR-based SRT data, with M/F relative levels (−9, −6, −3, 0, 3, 6, and 9 dB) as the factor. Results showed a significant effect for relative level [F(6, 66) = 11.522, p < 0.001]. *Post hoc* Bonferroni pairwise comparisons showed no significant difference between 0 dB and any M/F levels < 0 dB. Significant differences were observed between 0 dB and M/F levels > 0 dB (3 dB: p = 0.004; 6 dB: p < 0.001; 9 dB: p < 0.001).

## 4. Discussion

The present data provide further evidence that in competing speech, two-talker masker effectiveness is primarily determined by the masker stream that is most perceptually similar to the target. However, SRTs may also be affected by the relative levels of perceptually similar and dissimilar maskers.

An intelligible speech masker stream that has the same talker sex as the target is expected to be more perceptually similar to the target than would a masker stream with a different sex from the target (e.g., Kidd *et al.*, 2016), likely resulting in increased informational masking. The large difference in mean SRTs between the male and female maskers alone [Fig. 1(A)] suggests that the male masker was more perceptually similar to the target than was the female masker. The 8.8 dB masking release due to talker sex cues was consistent with previous studies (e.g., Cullington and Zeng, 2008: ∼9 dB; Liu *et al.*, 2021: ∼9 dB; Thomas *et al.*, 2021: ∼8 dB). When the male and female masker were combined at the same level (0 dB M/F level), the mean SRT was −2.9 dB SNR, much poorer than the one-talker SRT averaged across the male and female maskers (–15.9 dB). The 13 dB multi-talker penalty is generally consistent with our expectation and previous findings (e.g., Cullington and Zeng, 2008: ∼14 dB; Thomas *et al.*, 2020: ∼10 Db; Liu *et al.*, 2021: ∼15 dB; Chen *et al.*, 2020: ∼14 dB). The large performance difference between the one- and two-talker masker conditions is partly due to the additional informational masking associated with two competing talkers, when the maskers are at the same level (e.g., Freyman *et al.*, 2004; Rosen *et al.*, 2013). Increased energetic masking may also contribute to the performance difference between one-talker and two-talker maskers, as Thomas *et al.* (2021) found a significant difference in SRTs between one-talker and two-talker maskers when the informational masking was minimized with reversed speech.

According to our assumption, SRTs would be determined by the relative levels of target and the combined maskers (i.e., constant SNR-based SRTs) if the dissimilar masker mostly produced energetic masking; in this scenario, the relative levels of the two maskers would not influence the degree of informational masking. The data from the present study show that this assumption was only true at positive M/F masker levels. When the level of the perceptually similar masker is greater than that of the dissimilar masker, it is unlikely that the dissimilar masker will provide any additional informational masking since it is difficult to segregate the quieter dissimilar masker from the louder similar masker. Similar findings have been observed with two perceptually similar speech maskers. Brungart *et al.* (2001) found that speech performance depended almost exclusively on the overall SNR (i.e., the relative level between the target and combined maskers), rather than the relative masker levels when the two maskers were perceptually similar to the target.

Contrary to our assumption, SNR-based SRTs gradually improved with reduced M/F levels when the M/F level was ≤0 dB, suggesting that informational masking was reduced with decreased M/F levels. As shown in Fig. 3(A), TMR-based SRTs (relative to the perceptually similar masker sentence) were relatively constant across the negative M/F levels, suggesting that listeners could ignore the perceptually dissimilar masker, even though it was louder than the perceptually similar masker. Mean TMR-based SRTs ranged from 0.0 to −1.2 dB TMR when the M/F level was ≤0 dB, suggesting that listeners had difficulty segregating the target from the perceptually similar masker when the masker level was equal to or greater than that of the target. When there was a level difference between the two maskers, the level of the target talker at the SRT was generally in between the levels of the two maskers. As such, listeners could achieve negative TMRs with respect to the louder talker [see Fig. 3(C)] but were still affected by the level of the quieter talker during the adaptive-tracking procedure [see Fig. 3(D)].

Note that while the different M/F level conditions were randomized within and across listeners, the M/F level was fixed within a single test block. As such, listeners could predict the relative levels of the target and masker sentences on each trial within a block. These level cues may have allowed listeners to selectively attend to the target within test blocks (Brungart *et al.*, 2001; Byrne *et al.*, 2022). Such level cues can significantly affect speech performance for certain ranges of SNR. For one-talker maskers, SNR has relatively little effect on the intelligibility of the target sentence for SNRs between 0 and −10 dB (Brungart, 2001). This may affect the monotonicity criterion for adaptive up–down methods, especially for one-talker maskers or two-talker maskers when there is a substantial level difference between the maskers (Eddins and Liu, 2012), resulting in high variability in SRTs within and across subjects. Indeed, the largest variability in the present SRTs [standard deviation (SD) = 4.1 dB] was observed for the one-talker masker that was perceptually similar to the target, as predicted by Brungart (2001). With the two-talker maskers, the smallest variation (SD = 1.1 dB TMR) was observed when the M/F level was −9 dB. The data suggest that level cues may have little effect on the monotonicity of the adaptive procedure with two-talker maskers, even with varying relative levels, at least for the range used in the present study (−9 to 9 dB).

The mean SRT with the male masker alone was −11.5 dB TMR, much lower than the TMR-based SRTs for M/F levels ≤ 0 dB. When the M/F levels were >0 dB, mean TMR-based SRTs ranged from −1.8 to −3.1 dB, much higher than the mean SRT with the male masker alone. This provides further evidence of a multi-masker penalty, even when the relative levels vary between maskers, at least for the range used in the present study. The observed multi-masker penalty is not surprising as these level conditions generally met two conditions required for multi-masker penalty (Iyer *et al.*, 2010): (1) the stimulus contained at least one perceptually similar masker that could be confused with the target; and (2) the relative level of the target relative to both masker sentences was less than 0 dB [see Fig. 1(B)].

Note that the present M/F relative levels were relatively small (–9 dB to +9 dB). Within this range, TMR-based SRTs were unchanged when the level of the perceptually dissimilar masker was greater than that of the similar masker. SNR-based SRTs were unchanged when the level of the perceptually similar masker was greater than that of the dissimilar masker. However, this pattern may not hold for extreme masker level imbalances. For example, an extremely positive M/F level would effectively result in a one-talker male masker. The performance/intensity function is unclear for M/F levels >9 dB, where the maximum mean SRT for the present male masker would be −11.5 dB [Fig. 1(A)]. Linear extrapolation from the present data suggests that an approximately 30 dB M/F level would be required for SRTs with the one- and two-talker maskers to converge.
